# Outcome of Robot-Assisted Radical Cholecystectomy in a High-Volume Tertiary Cancer Center in India

**DOI:** 10.1089/vor.2018.0539

**Published:** 2019-10-17

**Authors:** Mahesh Goel, Sagar R. Kurunkar, Amol Kanetkar, Shraddha Patkar

**Affiliations:** GI and HPB Service, Department of Surgical Oncology, Tata Memorial Hospital, Mumbai, India.

**Keywords:** robotic radical cholecystectomy, gallbladder cancer, outcomes

## Abstract

***Introduction:*** Minimally invasive radical cholecystectomy is a complex laparoscopy. Robotic surgery is now an option to complete a radical cholecystectomy because of its high definition, magnified three-dimensional view of the operative field, and articulating instrumentation.^1–3^ Robotic surgery enables a safe dissection in otherwise difficult to access areas such as the porta hepatis. This video reviews the role of robotic surgery in the management of gall bladder (GB) malignancy.

***Methods:*** A 28-year-old lady, with no comorbidities, presented with abdominal pain and underwent an evaluation with a contrast-enhanced CT scan of chest and abdomen. The CT scan revealed a mass in the GB with no evidence of distant metastases. Liver function tests were normal and a CA19-9 was 898 U/mL. A robotic radical cholecystectomy using five ports (four robotic and one assistant port) was performed. The procedure started by clearing the hepatoduodenal ligament nodes (stations 8, 12, and 13 with interaortocaval node sampling). The triangle of Calot was then dissected and secured with clips. Next a wide excision of segments 4b and 5 was performed including the GB. The complete specimen was extracted in a bag from a small incision at the assistant port.

***Results:*** The procedure was performed in 330 minutes with a blood loss of 200 mL. There were no perioperative complications and the postoperative stay was 3 days. Final histopathology report revealed moderately differentiated adenocarcinoma of GB invading serosa (pT3) with negative margins and 4 out of 14 lymph nodes showed presence of metastases. The overall cohort shows 22 robotic radical cholecystectomies for GB malignancy. The median age was 53 years. The average duration of surgery was 270 minutes with a median blood loss of 120 mL. The median postoperative stay was 4 days and the median nodal yield for radical cholecystectomy was 8. The overall median survival at 18 months was 100% with one recurrent hepatic lesion.

***Discussion:*** Robotic radical cholecystectomy may offer technical superiority over laparoscopic surgery and is an oncologically acceptable approach with good short-term oncologic outcomes. This type of surgery may require a highly specialized center with adequate experience in hepatopancreatobiliary surgery.

*No competing financial interests exist.*

Runtime of video: 9 mins 5 secs

## Graphical abstract

**Figure:**
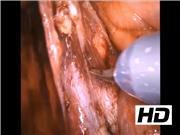

**Figure:**
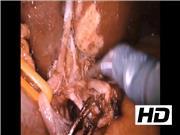

**Figure:**
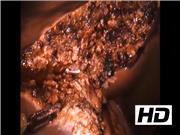

## Video

## Files

**MP4:** 

**PDF:** 
